# Motley, the Historian, Not a Surgeon

**Published:** 1861-07

**Authors:** 


MOTLEY, THE HISTORIAN, NOT A SURGEON.—In "The Rise of the Dutch Republic," by this gentleman, is an account of the attempted assassination of the Prince of Orange, whose escape is thus fancifully explained by the author:—

"On the 18th of March, 1582, the prince narrowly escaped with his life from the pistol of an assassin. While passing from the dining-room of the palace at Antwerp, he was met at the ante-chamber of his own apartments by a young man, who offered him a petition. The prince took the paper, and as he received it, the stranger suddenly drew a pistol, and discharged it at his head. The ball entered the neck under the right ear, and, passing through the roof of the mouth, came out under the left jaw. His hair and beard were set on fire by the discharge. He was believed to be mortally wounded by all who stood by. After recovering from the shock, his first words were, 'Do not kill him, I forgive him my death.' But his message of mercy was too late. Two of the gentlemen present had run the assassin through with their rapiers. The halberdiers rushed upon him in a moment, and he fell covered with mortal wounds. The prince was supported to his chamber by his friends, and, upon a surgical examination, the wound was found less dangerous than had been supposed. This was owing to a singular circumstance. The flame from the pistol had been so close that it had cauterized the wound inflicted by the ball. The flow of blood, which would otherwise have proved fatal, was thus prevented. The papers found upon the person of the assassin were all in the Spanish language, showing the Spanish origin of the plot, if any plot had existed. The pistol with which he had done the deed was lying upon the floor—a naked poniard was concealed in his clothes—in his pocket were various Catholic emblems and charms, a Jesuit catechism, a prayer book, Spanish bills of exchange to a considerable amount, and a set of writing tablets. These last were covered with inscriptions, relating to his murderous project, and showing the depth of superstition in which his mind was sunk. It was discovered that the assassin was in the employ of a Spanish merchant of Antwerp, who had been bribed by Philip to procure the death of the prince. Before the exposure of the plot, the merchant had made his escape, but two of his confederates were arrested, and, after a full confession, perished on the scaffold."

If Mr. Motley were acquainted with the nature of gunshot injuries, he would know that they seldom bleed profusely unless attended with the lesion of important vessels, that bullets often pursue a most extraordinary route without inflicting serious mischief, and that the explosion of gunpowder, although it may severely burn a wound, is not an efficient hæmostatic. The Prince of Orange evidently owed his life to the comparatively harmless route of the assassin's ball rather than to the cauterization of his wound.

